# Paleoecological evidence for decadal increase in phytoplankton biomass off northwestern Australia in response to climate change

**DOI:** 10.1002/ece3.3836

**Published:** 2018-01-18

**Authors:** Zineng Yuan, Dongyan Liu, John K. Keesing, Meixun Zhao, Shixin Guo, Yajun Peng, Hailong Zhang

**Affiliations:** ^1^ Key Laboratory of Coastal Zone Environmental Processes and Ecological Remediation Yantai Institute of Coastal Zone Research Chinese Academy of Sciences Yantai Shandong China; ^2^ Shandong Provincial Key Laboratory of Coastal Zone Environmental Processes Yantai Shandong China; ^3^ State Key Laboratory of Estuarine and Coastal Research East China Normal University Shanghai China; ^4^ CSIRO Oceans and Atmosphere Research University of Western Australia Oceans Institute Western Australian Marine Science Institution Indian Ocean Marine Research Centre Crawley WA Australia; ^5^ Key Laboratory of Marine Chemistry Theory and Technology Ocean University of China Ministry of Education Qingdao China; ^6^ Laboratory of Marine Ecology and Environmental Science Qingdao National Laboratory for Marine Science and Technology Qingdao China; ^7^ School of Management Science Guizhou University of Finance and Economics Guiyang Guizhou China

**Keywords:** biomarkers, Cygnet Bay, diatom, dinoflagellate, ocean warming, ${\text{TEX}}_{86}^{H}$

## Abstract

Ocean warming can modify the phytoplankton biomass on decadal scales. Significant increases in sea surface temperature (SST) and rainfall in the northwest of Australia over recent decades are attributed to climate change. Here, we used four biomarker proxies (TEX_86_ index, long‐chain *n*‐alkanes, brassicasterol, and dinosterol) to reconstruct approximately 60‐year variations of SST, terrestrial input, and diatom and dinoflagellate biomass in the coastal waters of the remote Kimberley region. The results showed that the most significant increases in SST and terrestrial input occurred since 1997, accompanied by an abrupt increase in diatom and dinoflagellate biomasses. Compared with the results before 1997, the average ${\text{TEX}}_{86}^{H}$ temperature during 1997–2011 increased approximately 1°C, rainfall increased 248.2 mm, brassicasterol and dinosterol contents increased 8.5 and 1.7 times. Principal component analysis indicated that the warming SST played a more important role in the phytoplankton increase than increased rainfall and river discharge.

## 1 INTRODUCTION

Phytoplankton is a key component in marine ecosystems, and its variations in abundance and species composition are sensitively coupled with short‐ and long‐term environmental changes, and consequently influence the structure and function of ecosystems, for example, biogeochemical cycles and the food web (Field, Behrenfeld, Randerson, & Falkowski, 1998). Over the last several decades, phytoplankton regime shifts, for example, the changes in biomass and species composition and shifts between diatom and nondiatom communities, have been widely observed in many coastal ecosystems (Smetacek & Cloern, 2008). Most evidence demonstrated that nutrient enrichment is a principal driving factor for phytoplankton regime shifts in coastal waters (Anderson, Glibert, & Burkholder, 2002), and ocean warming could enhance this process, affecting the distribution and productivity of phytoplankton in the ocean (Irwin, Finkel, Müller‐Karger, & Troccoli, 2015). For example, the warmer sea surface temperature (SST) and lower turbidity in the North Sea have increased phytoplankton biomass, even though nutrient concentrations have been decreasing since the 1980s (Mcquatters‐Gollop et al., 2007).

Due to limited observational data, it is challenging to distinguish between the impact of climatic variability and anthropogenic disturbances on the phytoplankton in coastal waters. Paleoecological methods, using geochemical and biological information preserved in the sediment to reconstruct the short‐ or long‐term environmental change, have supported significant findings in marine research, although a series of biological, chemical, and physical factors (e.g., water depth, temperature, salinity, grain size, and degradation) during sedimentation and preservation can impact the results (Fischer & Wefer, 1999). A few proxies have been widely applied to reconstruct sea temperature, terrestrial input, and phytoplankton biomass, due to their biosynthetic specificity and resistance to degradation in the sediment. Schouten, Hopmans, Schefuß, and Sinninghe Damsté (2002) proposed TEX_86_ (TetraEther indeX of tetraethers consisting of 86 carbon atoms) as a proxy for SST, based on the relative distribution of marine archaea isoprenoid glycerol dialkyl glycerol tetraethers (GDGTs). The selected GDGTs are membrane lipids synthesized by Thaumarchaeota, which contain different numbers of cyclopentane and cyclohexane rings. It has been demonstrated that the addition of rings into GDGTs enables archaea to adjust membrane stability in response to temperature changes (Chong, 2010; Uda, Sugai, Itoh, & Itoh, 2001). The long‐chain *n*‐alkanes (C_27_ + C_29_ + C_31_), specific to higher land plants, can help to interpret the impact of terrestrial input in marine sediments in terms of changes in rainfall, river discharge, or dust input (Eglinton & Hamilton, 1967; Seki et al., 2003). A few sterols are verified biomarkers for diatoms and dinoflagellates, for example, dinosterol (4α, 23, 24‐trimethyl‐5α‐cholest‐22(E)‐en‐3β‐ol) is produced almost exclusively by dinoflagellates, and brassicasterol (24‐methylcholest‐5, 22(E)‐dien‐3‐ol) is a commonly used diatom biomarker, even though it has been reported in many algal classes (Rampen, Abbas, Schouten, & Sinninghe Damste, 2010; Volkman et al., 1998). The analysis of these compounds in the sediment core can help to reconstruct the long‐term changes in the environmental change and phytoplankton community as well as their correlation.

**Figure 1:**
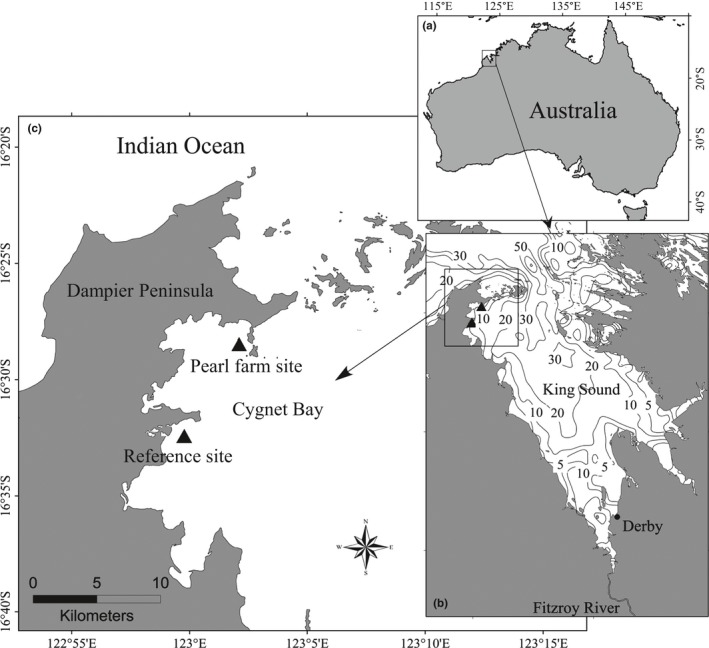
Study location: Cygnet Bay, northwestern Australia (a), water depth (m) in King Sound (b), and sampling site (c: reference site)

In this study, Cygnet Bay, located in the Kimberley, northwestern Australia (Figure 1a), was chosen to study the phytoplankton regime shift in response to the climate change. The Kimberley is a remote coastal region with very limited anthropogenic activity and significant climatic variability. During the past several decades, SST and rainfall have been reported as the most prominent climate‐induced changes in the northwest coast of Australia. For example, annual averaged SST has warmed by ca. 0.6°C in the past 50 year (Lough, 2008) and annual rainfall increased approximately 50% since 1950 (Feng, Li, & Xu, 2013; Shi et al., 2008). More recently, Furnas and Carpenter (2016) found that primary production in northern Australia increased more than twofold post‐1990 compared to the 1960s, but considering the paucity in data, they attributed the difference to the improvements in productivity measurements. Liu, Peng, Keesing, Wang, and Richard (2016) analyzed the variation in organic matter in the sediment cores, which were collected from Cygnet Bay, and they suggested that the variability in climatic signals (rainfall and temperature) might explain the increase in marine organic matter over decadal scales. Therefore, it warrants further examination to elucidate whether the increase in phytoplankton production in the northwest of Australia since the 1990s was related to climate‐induced SST and rainfall changes. Diatoms and dinoflagellates are the dominant phytoplankton in Kimberley coastal waters (Thompson & Bonham, 2011) thereby allowing us to use brassicasterol and dinosterol to represent diatom and dinoflagellate biomasses. Four proxies (TEX_86_ index, long‐chain *n*‐alkanes, brassicasterol, and dinosterol) were chosen and analyzed, using the dated sediment cores from Cygnet Bay, to reconstruct the variations of SST, terrestrial influence, and the diatom and dinoflagellate biomasses, respectively. Validity of biomarker reconstruction and the change in SST and phytoplankton over time are discussed in the context of historical observation data.

## 2 MATERIALS AND METHODS

### 2.1 Study area and core information

Cygnet Bay has an area of approximately 150 km^2^ and an average water depth of 10 m and is located in King Sound, which is the receiving water body of the Fitzroy River catchment (Figure 1b). Fitzroy River is the largest river discharging into King Sound and influencing Cygnet Bay, with 7035 GL of annual discharge and almost all the discharge occurs in the austral summer (data from Western Australian Department of Water, station: Fitzroy River‐Willare). Due to the impact of the semiarid tropical climatic regime, the evaporation rate is about 3,000 mm/year, greatly exceeding the rainfall, and the annual SST ranges from 26.1 to 30.3°C. The hydrodynamic processes in King Sound are mainly controlled by tide‐driven currents, and average tidal velocities are higher than 0.2 m/s (Wolanski & Spagnol, 2003). Only 3,261 persons live around Derby (Australian Bureau of Statistics 2011; www.censusdata.abs.gov.au; Figure 1b), and pearl oyster farming is the only anthropogenic activity in the bay (Figure 1c), spanning back approximately 50 years. Previous studies indicated that the environmental impact of this aquaculture activity was low due to the low stocking density and fast rate of water exchange (Jelbart, Schreider, & Macfarlane, 2011; Liu et al., 2016).

The cores used in this study were from the collection of Liu et al. (2016) in 2011, by SCUBA divers using a push core with a 6 cm internal diameter. Four replicate cores were taken at each site to enable a range of different parameters to be analyzed. The purpose of the study by Liu et al. (2016) was to investigate the impact of pearl farming on the sediment quality; hence, they chose one contrasting reference site 1.5 km away from the pearl aquaculture area (Figure 1c; reference site: 16°32′S, 122°59′E; water depth: 9.8 m). In this study, one of the cores (106 cm long) from the reference site was used for biomarker analyses (TEX_86_, long‐chain *n*‐alkanes, brassicasterol, and dinosterol). These biomarkers were not used in the previous study of Liu et al. (2016), as they focused on the analysis of chronology and geochemical parameters (organic matter, carbon and nitrogen isotopes, biogenic silica) to examine whether aquaculture had induced any change in the sediment over time. The replicate core used in this study had a similar sediment texture compared to other cores at the reference site, and the median grain size (d_50_) between replicate cores was significantly correlated at a 95% confidence level (Liu et al., 2016). The core we used covered a time span of approximately 1916–2011, according to the dating result (sedimentation rate: 1.11 cm/year) in Liu et al. (2016). This study focused on the data variations for the period 1940–2011, matching the observational data from Australian Bureau of Meteorology.

### 2.2 Chemical analysis of four biomarker proxies

The core used for biomarker analysis was sectioned at 1‐cm intervals and freeze dried prior to biomarker analysis. Sample processing and instrumental analyses of biomarker proxies were performed at Ocean University of China, using the same methods described in Li, Zhao, Tian, and Li (2013). Briefly, about 5 g of sediment was extracted four times with dichloromethane/MeOH (3:1, v/v), after adding internal standards (*n*‐C_24_D_50_, C_19_ *n*‐alkanol and C_46_ GDGT). Extracts were hydrolyzed with 6% KOH in MeOH. The neutral lipids were extracted with hexane and separated into two fractions using silica gel chromatography. The nonpolar lipid (containing *n*‐alkanes) fraction was eluted with hexane, and the polar lipid fraction (containing sterols and GDGTs) was eluted with dichloromethane/methanol (95:5, v/v). Subsequently, the polar fraction was divided into two parts, one derivatized using N, O‐bis (trimethylsilyl)‐trifluoroacetamide (BSTFA) at 70°C for 1 hr and the other filtered by PTFE membrane (0.45 μm) before instrumental measurements.

The long‐chain *n*‐alkanes, brassicasterol, and dinosterol were quantified by GC (Agilent 6890N) with an FID detector and a HP‐1 column (50 m × 0.32 μm × 0.17 μm). The oven temperature was programmed from 80°C for 1 min and then increased to 200°C at 25°C/min, followed by 4°C/min to 250°C, then 1.6°C/min to 300°C (holding for 12 min), 5°C/min to 320°C (holding for 5 min). GDGT analysis was performed by HPLC‐MS (Agilent 1200/Waters Micromass‐Quattro Ultima^TM^ Pt) with APCI probe and Prevail Cyano Column (150 × 2.1 mm, 3 μm). Separation was achieved with a flow rate of 0.3 ml/min at 30°C using a gradient program: 88% A (hexane) and 12% B (hexane/isopropanol, v/v = 9:1) at first, a linear gradient to 14% B in 3 min and to 24% B in 6 min; 76% A and 24% B for 5 min; a linear gradient to 100% B in 2 min; 100% B for 8 min; returning to 88% A and 12% B for 13 min. The analytical accuracy was better than 10% (Relative Standard Deviation) for these lipid contents in our laboratory.

The ${\text{TEX}}_{86}^{H}$ index, a modified version of TEX_86_, was calculated based on the relative abundance of GDGTs (Equation (1)) and converted into SST according to the global equation (Equation (2)), which is based on 255 core‐top dataset sediments with modern annual surface temperature (Kim et al., 2010). The analytical accuracy was better than 0.5 °C for ${\text{TEX}}_{86}^{H}$ temperature in our laboratory.

(1) $${\text{TEX}}_{86}^{H}=\text{log}\left(\frac{\left[\text{GDGT}\;2\right]+\left[\text{GDGT}\;3\right]+\left[{\text{Cren}}^{\prime }\right]}{\left[\text{GDGT}\;1\right]+\left[\text{GDGT}\;2\right]+\left[\text{GDGT}\;3\right]+\left[{\text{Cren}}^{\prime }\right]}\right)$$

(2) $$\text{SST}=68.4\times {\text{TEX}}_{86}^{H}+38.6,\;\;R^{2}=.87,\;\;n=255$$

where H stood for high temperature regions, the numbers 1–3 indicated the number of cyclopentane rings in GDGTs and Cren’ was the regioisomer of crenarchaeol.

The input of terrestrial isoprenoid GDGTs can impact the accuracy of ${\text{TEX}}_{86}^{H}$ in coastal waters. This error can be assessed by the Branched and Isoprenoid Tetraether (BIT) index, as ${\text{TEX}}_{86}^{H}$ is not suitable for SST reconstruction when BIT is above 0.3 (Weijers, Schouten, Spaargaren, & Sinninghe Damsté, 2006). Thus, we investigated the BIT index to evaluate the potential influence of soil‐derived GDGTs on ${\text{TEX}}_{86}^{H}$. The BIT index was calculated based on the relative abundance of branched GDGTs (Ia, IIa, and IIIa) and crenarchaeol (Cren) defined by Hopmans et al. (2004) (Equation (3)). The analytical accuracy was better than 0.006 for BIT index in our laboratory.

(3) $$\text{BIT}=\frac{\left[\text{GDGT‐Ia}]+[\text{GDGT‐IIa}]+[\text{GDGT‐IIIa}\right]}{\left[\text{GDGT‐Ia}]+[\text{GDGT‐IIa}]+[\text{GDGT‐IIIa}]+[\text{Cren}\right]}$$

### 2.3 Data source and statistical analysis

In order to verify the accuracy of ${\text{TEX}}_{86}^{H}$ reconstructed temperature, we used the extended reconstruction of SST (ERSST; resolution: 2°×2°; location: 16°S, 124°E; http://apdrc.soest.hawaii.edu/las/v6/constrain?var=286; Smith, Reynolds, Peterson, & Lawrimore, 2008) during the period of 1940–2011 as a comparison. To understand the impact of terrestrial input, we obtained the 1941–2011 rainfall data from the Australian Bureau of Meteorology (http://www.bom.gov.au/; Broome rainfall station) and the 1963–2011 river discharge data from Western Australian Department of Water (http://www.water.wa.gov.au/home ; Fitzroy River‐Dimond Gorge station).

The magnitudes of the shift changes of four biomarker proxies and observational data over time were assessed using the sequential *t* test analysis of regime shifts (STARS) (www.BeringClimate.noaa.gov; Rodionov, 2004; Rodionov & Overland, 2005). The cutoff length (*l*) was set to 10 year and the probability level to alpha = .05, representing a significant regime shift. STARS used a *t* test analysis to determine whether sequential records in a time series represent statistically significant (*p *<* *.05) departures from mean values observed during the preceding period of a predetermined duration (in our case, we used 10 years). After the shift point (date) was established, the magnitude change was reflected in the value of the regime shift index (RSI), which represents a cumulative sum of the normalized anomalies. The correlation between phytoplankton biomasses and environmental factors was analyzed using principal component analysis (PCA) using SPSS 16 (Statistical Package for the Social Sciences Inc.), and the eigenvalues were used to determine the fraction of total data variance explained by each principal component.

## 3 RESULTS

### 3.1 ${\text{TEX}}_{86}^{H}$ temperature record

**Figure 2:**
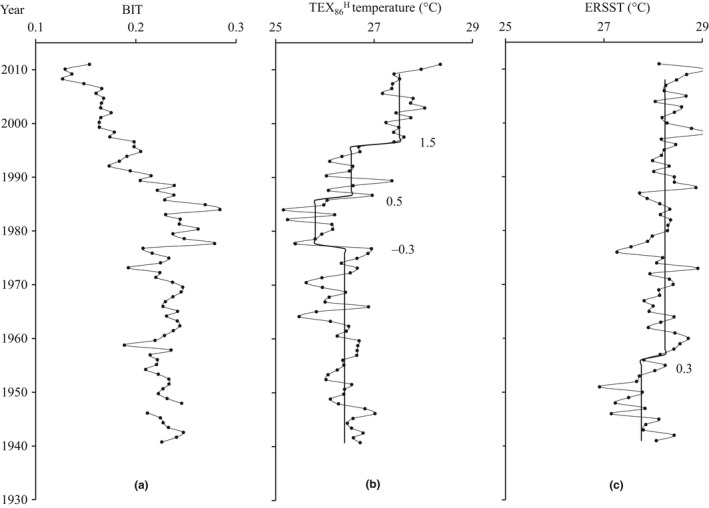
Profiles of BIT index (a) and sea temperature records (b: ${\text{TEX}}_{86}^{H}$ temperature; c: ERSST from 1940 to 2011). The solid lines show shift trends assessed by STARS, and numbers indicate regime shift indices

The range of BIT index (0.13–0.29) in our samples was generally lower than 0.3 (Figure 2a), indicating minimal influence from terrestrial GDGTs input and verifying the validity of the application of ${\text{TEX}}_{86}^{H}$ for SST reconstruction in Cygnet Bay. ${\text{TEX}}_{86}^{H}$ temperature ranged from 25.2 to 28.3°C and displayed three different periods according to the STARS analysis (Figure 2b): (1) 1940–1978 was relatively stable with an average of 26.4°C; (2) 1978–1986 showed a significant decrease with an average of 25.8°C (RSI: −0.3); (3) 1986–2011 displayed two significantly increasing shifts (RSI: 0.5, 1.5), and averages of 26.5°C (1986–1997) and 27.5°C (1997–2011), respectively. In comparison, ERSST ranged from 26.9 to 29.0°C (Figure 2c), which was slightly higher than ${\text{TEX}}_{86}^{H}$ temperature. The significant warming shift of ERSST occurred in 1956 (RSI: 0.5). The average temperatures of ERSST during 1940–1978, 1978–1986, and 1986–2011 were 27.9°C, 28.2°C, and 28.4°C, respectively. Thus, the increasing trend of SST during 1986–2011 is consistent between the two parameters.

### 3.2 Long‐chain n‐alkanes record

**Figure 3:**
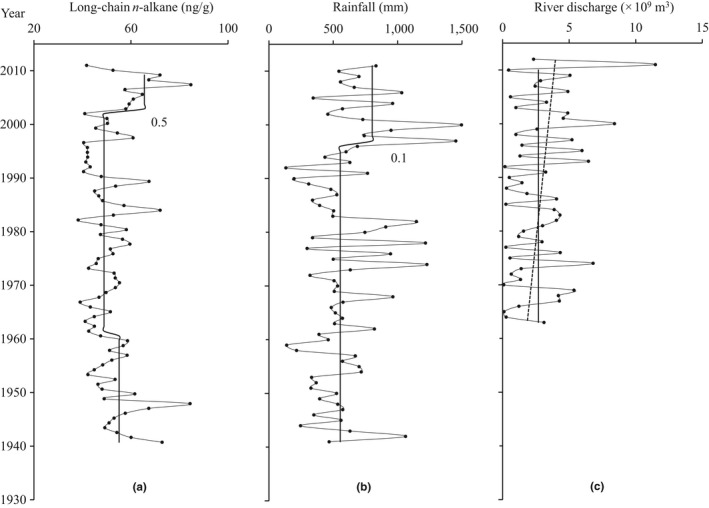
Profiles of terrestrial records: long‐chain *n*‐alkane contents (a), annual rainfall from 1941 to 2011 (b), and Fitzroy River discharge from 1963 to 2011 (c). The dashed line in Fitzroy River discharge shows the linear trend. The solid lines show shift trends from STARS, and numbers indicate regime shift indices

The contents of long‐chain *n*‐alkanes in marine sediments can help to interpret the variations of terrestrial input. They ranged from 38.2 to 84.7 ng/g and showed two periods over time according to STARS analysis (Figure 3a): (1) 1940–2002 was relatively stable with an average of 51.0 ng/g, although STARS detected a small decreasing shift (RSI < 0.1) in 1960; (2) 2002–2011 displayed higher values, with an average of 65.5 ng/g, with one significant increasing shift (RSI: 0.5) in 2002.

Figure 3b shows Broome rainfall ranged from 132 to 1,496 mm during 1941–2011. STARS separated them into two periods with a small increasing shift in 1997 (RSI: 0.1). The average rainfall during 1997–2011 was 802.2 mm, significantly higher than the average rainfall during 1941–1996 (553.7 mm). In comparison, STARS did not detect a significant shift in annual river discharge of the Fitzroy River during 1963–2011, despite a slightly increasing trend after the 1990s (Figure 3c). The average annual river discharge during 1997–2011 was 3.92 × 10^9^ m^3^/year, higher than during 1963–1996 (2.53 × 10^9^ m^3^/year). Thus, the patterns of long‐chain *n*‐alkanes, rainfall, and river discharge are broadly consistent in this region, indicating an increase after the late 1990s.

### 3.3 Phytoplankton biomarker records

**Figure 4:**
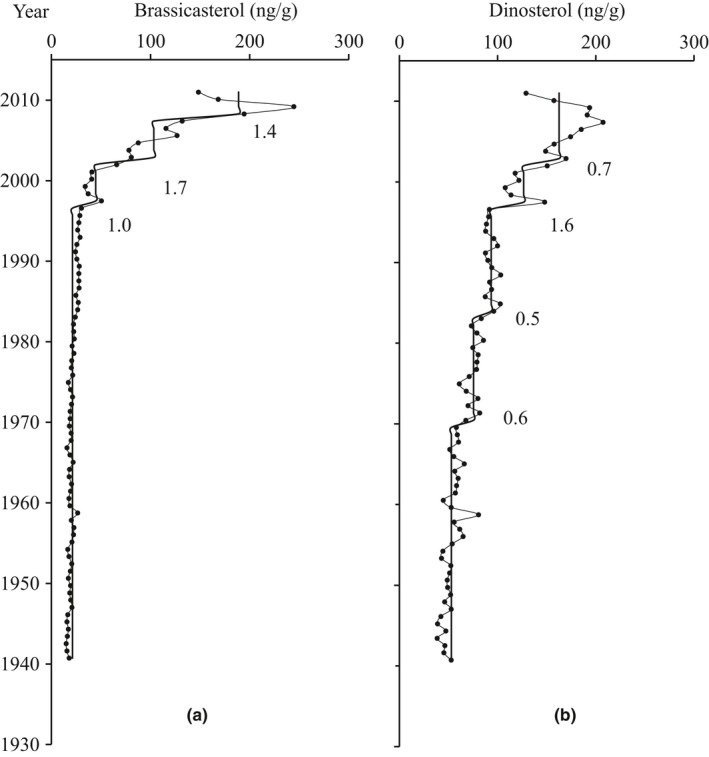
Profiles of phytoplankton records: brassicasterol contents (a) and dinosterol contents (b). The solid lines show shift trends assessed by STARS, and numbers indicate regime shift indices

The brassicasterol contents of the sediment, representing diatom biomass, varied from 14.8 to 244.6 ng/g with two significant periods (Figure 4a): (1) 1940–1997, brassicasterol contents were lower and stable, averaging 22.2 ng/g; (2) 1997–2011, brassicasterol contents increased rapidly, STARS detected three significant increases (RSI: 1.0, 1.7, and 1.4), and the average contents during these shifts were 44.7, 103.4, and 188.8 ng/g, respectively.

The dinosterol contents, representing dinoflagellate biomass, varied from 38.7 to 207.3 ng/g with two significant periods (Figure 4b): (1) 1940–1970, dinosterol contents were relative stable, averaging 52.9 ng/g; (2) 1970–2011, dinosterol contents increased gradually during 1970–1997 (RSI: 0.6 and 0.5; average: 75.6 and 93.5 ng/g) and more rapidly during 1997–2011 (RSI: 1.6 and 0.7; average: 126.6 and 162.7 ng/g). These results are consistent with the increased temperature and terrestrial input.

**Figure 5:**
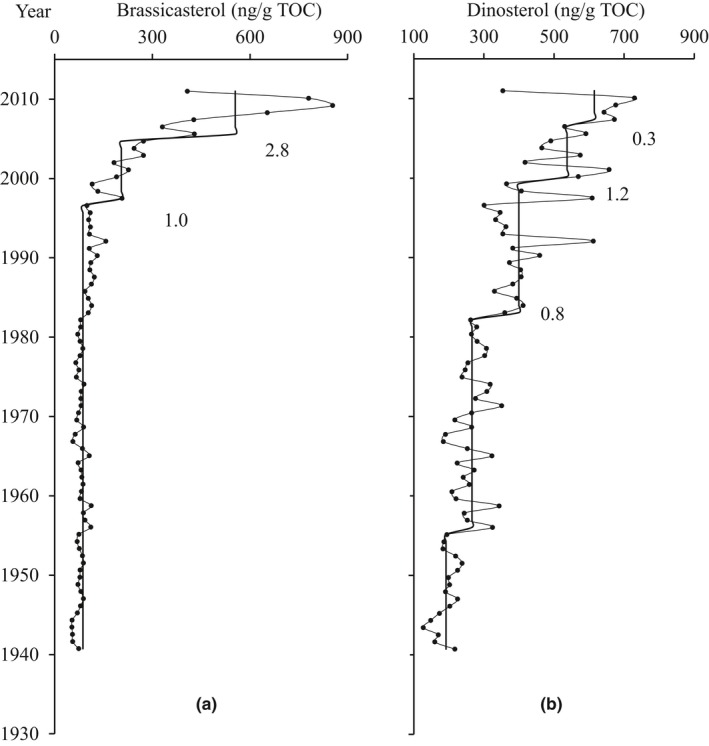
Profiles of phytoplankton records: TOC‐normalized brassicasterol contents (a) and TOC‐normalized dinosterol contents (b). The solid lines show shift trends assessed by STARS, and numbers indicate regime shift indices

TOC‐normalized biomarker content (e.g., nanogram of biomarker per gram of TOC in sediment) assists in evaluating the effect of variation in biomarker preservation in sediment as it can indicate the relative enrichment or depletion of any specific biomarker in comparison with the bulk TOC. The TOC‐normalized brassicasterol varied from 53.1 to 853.8 ng/g TOC with two significant periods (Figure 5a): (1) 1940–1997, brassicasterol contents were lower and stable, averaging 86.6 ng/g TOC; (2) 1997–2011, brassicasterol contents increased rapidly, STARS detected two significant increases (RSI: 1.0 and 2.8), and the averages during these shifts were 204.8 ng/g TOC and 554.7 ng/g TOC, respectively.

The TOC‐normalized dinosterol content varied from 127.4 to 729.7 ng/g TOC with two significant periods (Figure 5b): (1) 1940–1982, dinosterol contents were relative stable, averaging 237.8 ng/g TOC; (2) 1982–2011, dinosterol contents increased gradually during 1982–1999 (RSI: 0.8; average: 399.6 ng/g TOC) and more rapidly during 1999–2011 (RSI: 1.2 and 0.3; average: 537.1 ng/g TOC and 614.9 ng/g TOC). Thus, the temporal variations of TOC‐normalized biomarker contents were similar to those of the corresponding biomarker contents, suggesting minimal influence from variability in preservation.

### 3.4 Principal component analysis (PCA)

PCA of the four proxies showed the first two principal components (PC1 and PC2) were responsible for 82.8% of total variance. PC1 accounted for 57.7% of total variance, with high positive loadings on phytoplankton biomarkers brassicasterol (0.92) and dinosterol (0.92) and ${\text{TEX}}_{86}^{H}$ temperature (0.78). In addition, ${\text{TEX}}_{86}^{H}$ temperature showed significant correlations with phytoplankton biomarkers (coefficient of 0.56 for brassicasterol and 0.55 for dinosterol, *p *<* *.01), indicating the importance of SST on the phytoplankton biomass increase. PC2 accounted for 25.1% of the total variance, with a high positive loading for long‐chain *n*‐alkanes contents (0.99). In comparison, long‐chain *n*‐alkanes displayed poor correlations with phytoplankton biomarkers (brassicasterol: *r* = .02, *p *>* *.05; dinosterol: *r* = −.09, *p *>* *.05).

## 4 DISCUSSION

### 4.1 Validity of reconstructed proxies

${\text{TEX}}_{86}^{H}$ is suitable for SST reconstruction in tropical or subtropical regions (>15°C; Kim et al., 2012) and has been applied in the Australian region displaying a good linear correlation with instrumental annual SST (Chen, Mohtadi, Schefuß, & Mollenhauer, 2014; Smith et al., 2013). The warming trend of ${\text{TEX}}_{86}^{H}$ and ERSST in Cygnet Bay is similar, but there are some discrepancies in the warming rate (Figure 2). This phenomenon was reported in previous studies, for example, northwest Africa (Mcgregor, Dima, Fischer, & Mulitza, 2007), northeast Hong Kong (Kong et al., 2015), and the shelf of Western Australia (Zinke et al., 2015). A few factors could lead to the difference between ${\text{TEX}}_{86}^{H}$ temperature and ERSST. One is the errors from the measurement of ERSST as (1) sporadic SST measurements over the historical period can result in large uncertainties in ERSST (Kennedy, 2014; Smith et al., 2008); (2) the 2° × 2° resolution of ERSST could underestimate temperature variability in shallow waters, because the rapid warming rate was more frequently observed in the nearshore than offshore (Lima & Wethey, 2012). Other possible errors are from ${\text{TEX}}_{86}^{H}$ index: (1) The ${\text{TEX}}_{86}^{H}$ temperature using the global calibration has a large calibration error (2.5°C), affecting the accuracy of ${\text{TEX}}_{86}^{H}$ temperature; (2) some studies show the ${\text{TEX}}_{86}^{H}$ temperature may be skewed due to seasonality in growth or export of Thaumarchaeota (Jia, Zhang, Chen, Peng, & Zhang, 2012; Leider, Hinrichs, Mollenhauer, & Versteegh, 2010). In general, ${\text{TEX}}_{86}^{H}$ captured the warming trend in northwest Australia. Observed air and sea temperatures revealed significant warming since the 1950s and accelerated warming rates since the 1980s (Lough, 2008; Lough & Hobday, 2011). An increase in anomalously warm ocean conditions along the western Australian coastline has been recorded since the late 1990s, strongly influenced by the strengthening of the Indonesian Throughflow in response to increases in Pacific trade winds (Feng et al., 2011, 2015). Coral temperature records from northwestern Australia also indicated long‐term warming of coastal waters with the highest temperature anomalies recorded during the 1980s‐2010 (Zinke et al., 2015). These similar warming patterns therefore confirm the applicability of ${\text{TEX}}_{86}^{H}$ index in our study area.

Long‐chain *n*‐alkanes have been widely used to evaluate the terrestrial influence on the ocean (Eglinton & Eglinton, 2008). In this study, the increasing trend of long‐chain *n*‐alkanes during 2002–2011 matches the increased rainfall and river discharge after 1997, although there is a small time lag (Figure 3). Meanwhile, rapid increases in brassicasterol and dinosterol after 1997 coincided with increased ${\text{TEX}}_{86}^{H}$ temperature and long‐chain *n*‐alkanes in timescale (Figure 4). Liu et al. (2016) discovered a significant increase in total organic carbon and nitrogen in sediment cores in the Cygnet Bay after 2000; the C:N ratio and δ^13^C indicated the increased organic matter was mainly from marine sources. Furnas and Carpenter (2016) found primary production increased ca. twofold in 1990–2013 compared to 1960–1962 in the Kimberley. Thus, there is credible evidence for phytoplankton biomass increase related to climate change in the Kimberley shelf region.

### 4.2 Phytoplankton biomass in response to warming SST and terrestrial influence

Temperature, salinity, irradiance, and macronutrient concentrations are regarded as the fundamental environmental factors determining phytoplankton niches. Recently, statistical analysis of time series data in the Baltic Sea and Caribbean Sea showed that the importance of temperature, salinity, and irradiance for the niches of diatoms and dinoflagellates is even higher than macronutrient concentrations (Gasiūnaitė et al., 2005; Irwin, Nelles, & Finkel, 2012; Mutshinda, Finkel, & Irwin, 2013). In this study, a significant concurrent shift occurred in 1997. Compared with the average values of four proxies before 1997, the average ${\text{TEX}}_{86}^{H}$ temperature during 1997–2011 increased approximately 1°C, rainfall increased 248.2 mm, and brassicasterol and dinosterol sediment contents increased 8.5 and 1.7 times, respectively. The PCA indicated that warming temperature has a more significant impact on the increases in diatom and dinoflagellate biomass than terrestrial input. The driving mechanism of warming temperature on phytoplankton biomass is complicated as most results from open sea showed that ocean warming can enhance the vertical stratification, which could reduce the nutrient supply to the mixed‐layer and consequently reduce phytoplankton biomass in the surface water (Behrenfeld et al., 2006; Richardson & Schoeman, 2004). However, some studies indicated that warming temperature can accelerate nutrient recycling by bacteria, resulting in phytoplankton increase (Taucher & Oschlies, 2011). In Cygnet Bay, the seawater is well mixed due to the shallow depth (9.8 m) and strong tidal action all year round. Thus, the increase in phytoplankton biomass was more possibly attributed to positive physiological action and fast nutrient turnover. Some studies in northwestern Australia emphasized a mechanism of ocean‐coast interaction that could influence the coastal phytoplankton biomass, for example, in the North West Cape, the increase of phytoplankton biomass in coastal waters could be caused by increased nutrients transport from the deep sea through enhanced upwelling (Furnas, 2007). In Darwin Harbour, Burford, Alongi, Mckinnon, and Trott (2008) found that the oceanic inputs of nutrients to the estuary were a primary contributor. However, due to a lack of observational data, the mechanism of the ocean‐coast interaction in the Kimberley needs further exploration.

The sediment core analysis revealed not only the increased phytoplankton biomass since 1997 but also the different shifting pattern between diatoms and dinoflagellates. Dinoflagellates showed an earlier but slower increasing trend compared to diatoms (Figure 4). Thompson and Bonham (2011) analyzed the phytoplankton communities during a 2010 research voyage in the Kimberley and found the pigment proportion of diatoms in shallow water (<50 m) was much higher than for dinoflagellates. This supports our result in the upper section of the core. In general, diatoms are favored in niches with higher macronutrients, higher turbulence, lower salinity, and lower temperature than the dinoflagellates, and thus, diatoms often dominate in the coastal and estuarine waters (Berdalet, 1997; Margalef, 1978). Silicate is a critical, limiting macronutrient for diatom growth but not for dinoflagellates, and the source of silicate in coastal waters is mainly through riverine input. Thus, increased riverine input and rainfall often enhance the supply of silicate and decrease the salinity which can provide more suitable conditions for diatom growth. In this study, diatom and dinoflagellate biomarkers did not display any correlation with long‐chain *n*‐alkane (proxy for terrestrial inputs); however, the increased river discharge and rainfall after 1997 provide a mechanism by which conditions after that time favored diatoms over dinoflagellates.

In summary, the paleoecological evidence from Cygnet Bay demonstrated that SST and terrestrial input have significantly increased since 1997 in the Kimberley region, and the biomasses of diatoms and dinoflagellates corresponded to these changes with an increasing trend. In comparison, warming SST played a more important role for the phytoplankton increase than increased rainfall and river discharge.

## CONFLICT OF INTEREST

None declared.

## AUTHORS’ CONTRIBUTION

Zineng Yuan contributed to interpretation of data and drafting the work; Dongyan Liu contributed to the conception of the work, revising the draft and final approval of the version to be published; John Keesing contributed to design of the work and acquisition of samples; Meixun Zhao contributed to revising the draft and analysis methods; Shixin Guo contributed to the sample analysis; Yajun Peng contributed to interpretation of data; Hailong Zhang contributed to the sample analysis.
